# CTNNB1 Signaling in Sertoli Cells Downregulates Spermatogonial Stem Cell Activity via WNT4

**DOI:** 10.1371/journal.pone.0029764

**Published:** 2012-01-12

**Authors:** Alexandre Boyer, Jonathan R. Yeh, Xiangfan Zhang, Marilène Paquet, Aurore Gaudin, Makoto C. Nagano, Derek Boerboom

**Affiliations:** 1 Animal Reproduction Research Centre (CRRA), Faculté de Médecine Vétérinaire, Université de Montréal, St-Hyacinthe, Québec, Canada; 2 Department of Obstetrics and Gynecology, McGill University, Montréal, Québec, Canada; 3 Comparative Medicine and Animal Resources Centre, McGill University, Montréal, Québec, Canada; Clermont Université, France

## Abstract

Constitutive activation of the WNT signaling effector CTNNB1 (β-catenin) in the Sertoli cells of the *Ctnnb1* ^tm1Mmt/+^;*Amhr2* ^tm3(cre)Bhr/+^ mouse model results in progressive germ cell loss and sterility. In this study, we sought to determine if this phenotype could be due to a loss of spermatogonial stem cell (SSC) activity. Reciprocal SSC transplants between *Ctnnb1* ^tm1Mmt/+^;*Amhr2* ^tm3(cre)Bhr/+^ and wild-type mice showed that SSC activity is lost in *Ctnnb1* ^tm1Mmt/+^;*Amhr2* ^tm3(cre)Bhr/+^ testes over time, whereas the mutant testes could not support colonization by wild-type SSCs. Microarray analyses performed on cultured Sertoli cells showed that CTNNB1 induces the expression of genes associated with the female sex determination pathway, which was also found to occur in *Ctnnb1* ^tm1Mmt/+^;*Amhr2* ^tm3(cre)Bhr/+^ testes. One CTNNB1 target gene encoded the secreted signaling molecule WNT4. We therefore tested the effects of WNT4 on SSC-enriched germ cell cultures, and found that WNT4 induced cell death and reduced SSC activity without affecting cell cycle. Conversely, conditional inactivation of *Wnt4* in the *Ctnnb1* ^tm1Mmt/+^;*Amhr2* ^tm3(cre)Bhr/+^ model rescued spermatogenesis and male fertility, indicating that WNT4 is the major effector downstream of CTNNB1 responsible for germ cell loss. Furthermore, WNT4 was found to signal via the CTNNB1 pathway in Sertoli cells, suggesting a self-reinforcing positive feedback loop. Collectively, these data indicate for the first time that ectopic activation of a signaling cascade in the stem cell niche depletes SSC activity through a paracrine factor. These findings may provide insight into the pathogenesis of male infertility, as well as embryonic gonadal development.

## Introduction

Spermatogonial stem cells (SSCs) are the progenitor population of male germ cells. Similar to other stem cell types, they can be directed to one of two cell fate decisions: either self-renewal to maintain the SSC pool, or differentiation into more specialized germ cells that will eventually become spermatozoa. SSCs reside in a specialized microenvironment, termed the SSC niche, that controls the activity of SSCs and is believed to be formed mainly by Sertoli cells and the basal membrane along the vascular network [1], [2]. The Sertoli cells seem to be a particularly important component of the SSC niche, as numerous factors such as glial cell-derived neurotrophic factor (GDNF), fibroblast growth factor 2 (FGF2), kit ligand (KITL), activin A and bone morphogenic protein 4 (BMP4) are all produced by Sertoli cells and affect self-renewal, proliferation and differentiation of SSCs [3]–[7].

The wingless-related MMTV integration site (WNT) gene family encodes a large number of secreted signaling glycoproteins that are involved in many biological processes including embryonic development [8], [9], adult tissue homeostasis [10], maintenance of progenitor cell types [11] and cell fate determination and differentiation [12]–[15]. WNT signal transduction can occur via at least three distinct pathways, commonly referred to as the WNT/Ca^2+^, planar cell polarity and WNT/CTNNB1 or canonical pathway. In the latter, a pool of CTNNB1 (β-catenin) protein localizes to the cytoplasm, where it is resides in a large multiprotein complex that notably includes the scaffold proteins APC and AXIN. Bound to these proteins, CTNNB1 is rapidly phosphorylated by other components of the complex, but mainly by glycogen synthase kinase 3β. These phosphorylations result in the subsequent ubiquitination of the protein and its degradation by the cellular proteosomal machinery, and as a result there is little or no free CTNNB1 in the cytoplasmic pool in the resting state. The WNT/CTNNB1 pathway is activated by the binding of a WNT to a cognate receptor of the Frizzled (FZD) family, and ultimately results in CTNNB1 escaping the complex in a hypophosphorylated state, allowing it to accumulate within the cell and to translocate to the nucleus. In the nucleus, CTNNB1 associates with different transcription factors to modulate the transcriptional activity of various target genes in a cell type- and developmental stage-specific manner [8], [15]–[16]. WNT signaling in the postnatal testis has not been well studied, but it has been suggested to affect normal spermatogenesis. Notably, mice bearing a null mutation of the WNT signaling antagonist *Nkd1* have lower numbers of haploid spermatids [17], and WNT5a has been shown to promote SSC self-renewal [18]. Recently, ourselves and others [19]–[20] generated transgenic mice (*Ctnnb1* ^tm1Mmt/+^;*Amhr2* ^tm3(cre)Bhr/+^) in which Cre-lox recombination leads to the expression of a dominant-stable mutant of CTNNB1 in Sertoli cells, thereby activating the WNT/CTNNB1 pathway constitutively in these cells. These mice were sterile due to testicular atrophy associated with degeneration of the seminiferous epithelium starting by 5 wks of age and resulting in complete loss of germ cells before 4 months. *Ctnnb1* ^tm1Mmt/+^;*Amhr2* ^tm3(cre)Bhr/+^ Sertoli cells also exhibited morphological characteristics and gene expression patterns suggestive of incomplete differentiation that appeared in a manner coincident with germ cell loss. These data suggested that the WNT/CTNNB1 pathway disrupts Sertoli cell functions critical to their capacity to support spermatogenesis in the postnatal testis.

In the present study, we tested the hypothesis that the loss of spermatogenesis in *Ctnnb1* ^tm1Mmt/+^;*Amhr2* ^tm3(cre)Bhr/+^ animals results from defective Sertoli cells that cannot support SSC activity. We discovered that *Ctnnb1* ^tm1Mmt/+^;*Amhr2* ^tm3(cre)Bhr/+^ Sertoli cells lose their SSC niche capacity and that this is due to the ectopic expression of WNT4, which acts in a paracrine manner to downregulate SSC activity.

## Results

### *Ctnnb1^tm1Mmt/+^*;*Amhr2^tm3(cre)Bhr/+^* Sertoli cells fail to support spermatogonial stem cell activity

**Figure 1:**
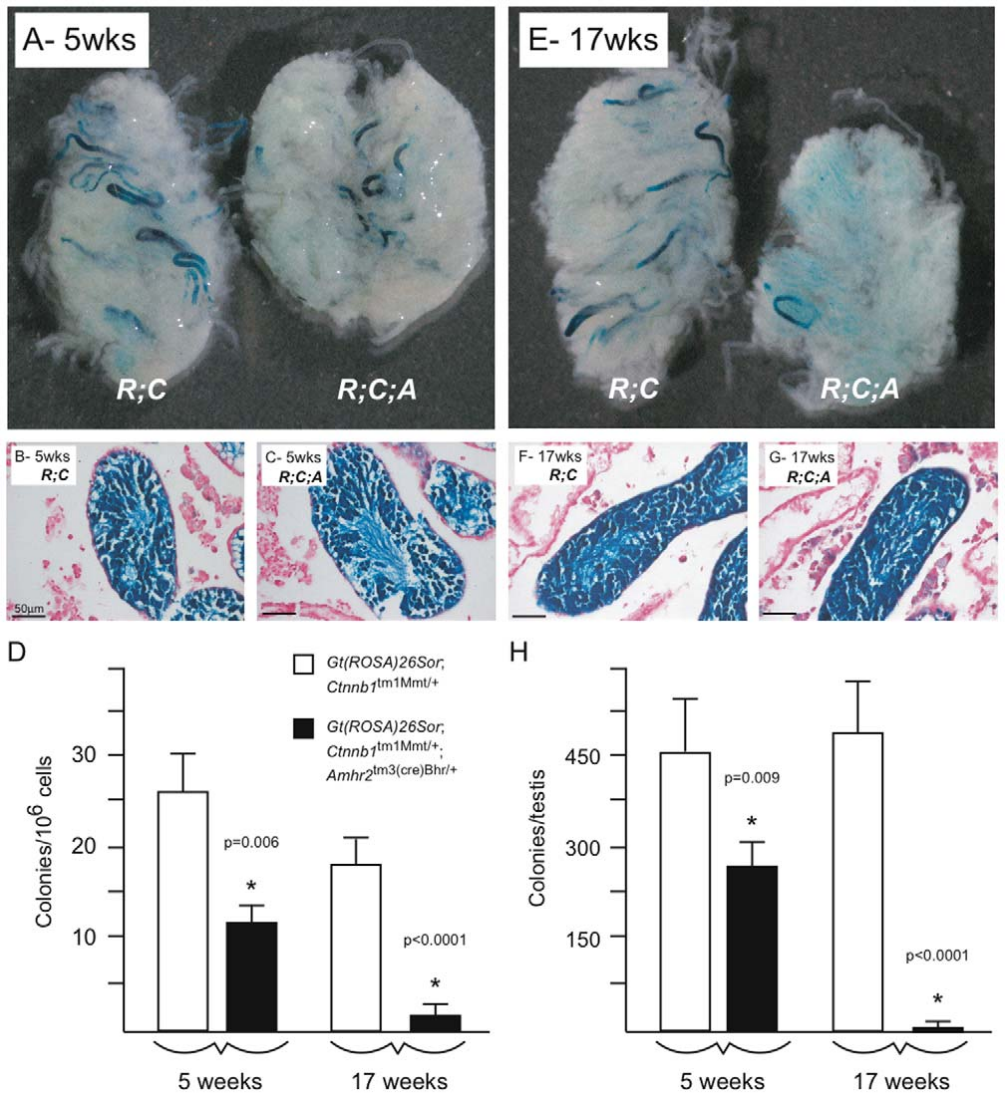
*Ctnnb1* ^tm1Mmt/+^;*Amhr2* ^tm3(cre)Bhr/+^ mice lose spermatogonial stem cell activity over time. (*A*, *E*) Photographs of decapsulated, LacZ-stained recipient testes 8 weeks after transplantation of donor cells from 5- (*A*) or 17- (*E*) week-old *Gt(ROSA)26Sor;Ctnnb1* ^tm1Mmt/+^;*Amhr2* ^tm3(cre)Bhr/+^ (*R*;*C*, control) and *Gt(ROSA)26Sor;Ctnnb1* ^tm1Mmt/+^;*Amhr2* ^tm3(cre)Bhr/+^ (*R*;*C*;*A*) mice. Original magnification 12.5×. (*B*, *C*, *F*, *G*) Photomicrographs demonstrating complete regeneration of spermatogenesis in the testes shown in (*A*) and (*E*). (*D*) Spermatogenic colony numbers obtained after donor cell transplantation, n = 8–9/time/genotype. Results are expressed as colonies per million transplanted cells. (*H*) Total spermatogonial stem cells present in the donor testes, calculated by multiplying colony numbers (*D*) by the total number of germ cells harvested from the donor testis. All data are expressed as mean (columns) ± SEM (error bars). Significant differences from controls (*P*<0.05) are indicated with an asterisk (*) and accompanied by relevant *P* values.

To determine if germ cell loss in the *Ctnnb1* ^tm1Mmt/+^;*Amhr2* ^tm3(cre)Bhr/+^ model could be associated with a loss of SSC activity, reciprocal SSC transplant studies were performed. In the first experiment, SSCs in mutant testes were transplanted into testes of germ cell-depleted wild-type recipient mice. To genetically label mutant donor cells with lacZ, *Ctnnb1* ^tm1Mmt/+^;*Amhr2* ^tm3(cre)Bhr/+^ mice were crossed with *Gt(ROSA)26Sor* mice. *Gt(ROSA)26Sor* mice express the *lacZ* transgene in virtually all cell types, including male germ cells, allowing the discrimination of donor cells from recipient cells after transplantation *in vivo*. Donor SSCs from 5 and 17 week-old *Gt(ROSA)26Sor;Ctnnb1* ^tm1Mmt/+^;*Amhr2* ^tm3(cre)Bhr/+^ and control *Gt(ROSA)26Sor;Ctnnb1* ^tm1Mmt/+^ mice were transplanted and the recipient testes analyzed two months later. A 56% reduction in the SSC frequency was observed in 5 week-old mutant mice (Fig. 1A–1D), corresponding to a 46% reduction in total functional SSC numbers per testis (Fig. 1H). SSC frequency further declined to 8.6% of control in testes of 17 week-old mutant mice, with total SSC numbers per testis reduced to 1% of control (Fig. 1D–1H). These results indicate that a loss of SSC activity occurs in *Ctnnb1* ^tm1Mmt/+^;*Amhr2* ^tm3(cre)Bhr/+^ mice in a manner temporally coincident with testicular atrophy and germ cell loss [19].

**Figure 2:**
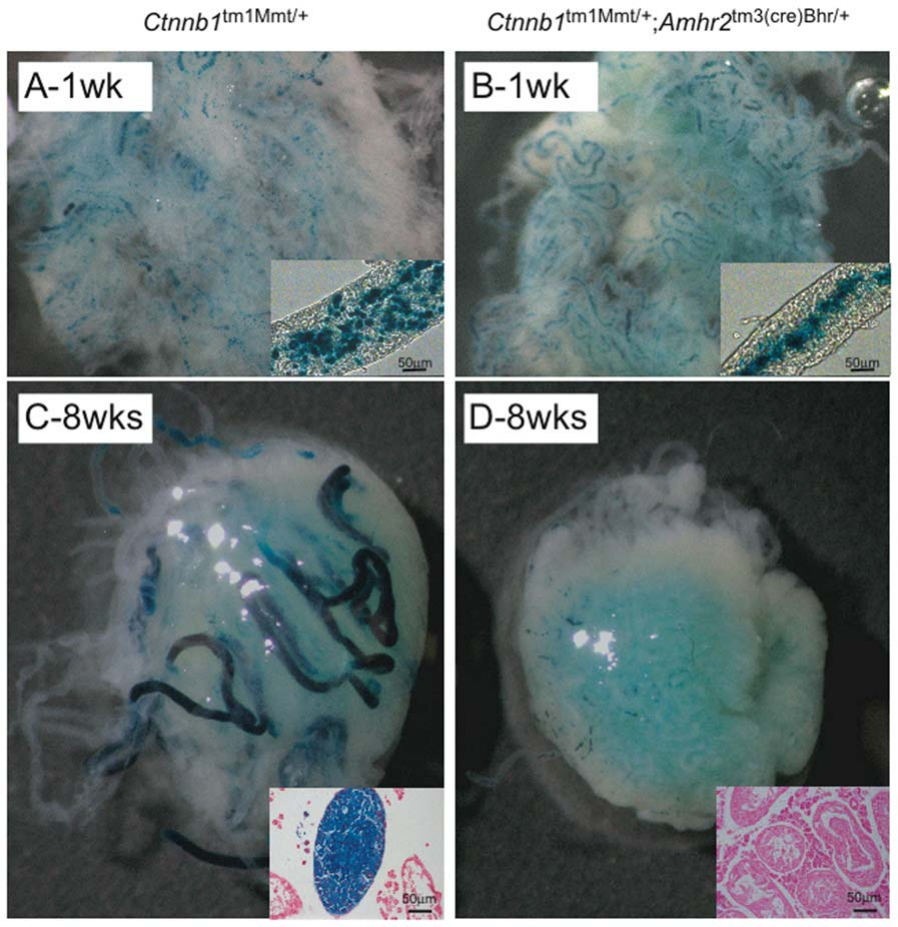
*Ctnnb1* ^tm1Mmt/+^;*Amhr2* ^tm3(cre)Bhr/+^ testes are unable to support donor SSCs. (*A*, *B*) Photographs of decapsulated, LacZ-stained recipient *Ctnnb1^tm1Mmt/+^* (*A*) or *Ctnnb1* ^tm1Mmt/+^;*Amhr2* ^tm3(cre)Bhr/+^ (*B*) testes 1 week after transplantation of *Gt(ROSA)26Sor* germ cells. Insets show higher magnification lateral views of seminiferous tubules from the corresponding testes. (*C*, *D*) Photographs of decapsulated, LacZ-stained recipient *Ctnnb1^tm1Mmt/+^* (*C*) or *Ctnnb1* ^tm1Mmt/+^;*Amhr2* ^tm3(cre)Bhr/+^ (*D*) testes 8 weeks after transplantation of *Gt(ROSA)26Sor* germ cells. Insets are photomicrographs demonstrating complete regeneration of spermatogenesis in the *Ctnnb1^tm1Mmt/+^* testes (*C*), whereas no evidence for spermatogenesis was detected in the *Ctnnb1* ^tm1Mmt/+^;*Amhr2* ^tm3(cre)Bhr/+^ testes (*D*). Original magnification 32× (*A*, *B*) or 16× (*C*, *D*).

In the second experiment, the ability of *Ctnnb1* ^tm1Mmt/+^;*Amhr2* ^tm3(cre)Bhr/+^ testes to support wild-type SSCs was examined by using 6-week-old mutant mice as recipients. Any endogenous germ cells remaining in recipient testes at this age were depleted, and *Gt(ROSA)26Sor* donor cells were transplanted into these testes. One week after transplantation, donor germ cells were present in both *Ctnnb1* ^tm1Mmt/+^;*Amhr2* ^tm3(cre)Bhr/+^ and control *Ctnnb1* ^tm1Mmt/+^ testes but differed in appearance (Fig. 2A, 2B). In control testes, donor cells were found on the basal membrane of the tubules, indicating that the cells had migrated from the lumen and colonized the recipient testes (Fig. 2A, inset). In contrast, no migrating cells were observed in the testis of *Ctnnb1* ^tm1Mmt/+^;*Amhr2* ^tm3(cre)Bhr/+^ mice, and most donor cells remained in the lumen (Fig. 2B, inset). When analyzed two months after transplantation, a complete regeneration of spermatogenesis was observed in control animals (24.4±2.5 colonies/10^6^ cells transplanted), whereas no donor-derived spermatogenesis was observed in *Ctnnb1* ^tm1Mmt/+^;*Amhr2* ^tm3(cre)Bhr/+^ testes (Fig. 2C, 2D). Taken together, these results suggest that the activation of CTNNB1 in Sertoli cells altered their functional properties, resulting in a failure of the SSC niche and the loss of the ability to support SSC activity.

### Sertoli cells in *Ctnnb1^tm1Mmt/+^*;*Amhr2^tm3(cre)Bhr/+^* testes express markers of female sex determination

**Figure 3:**
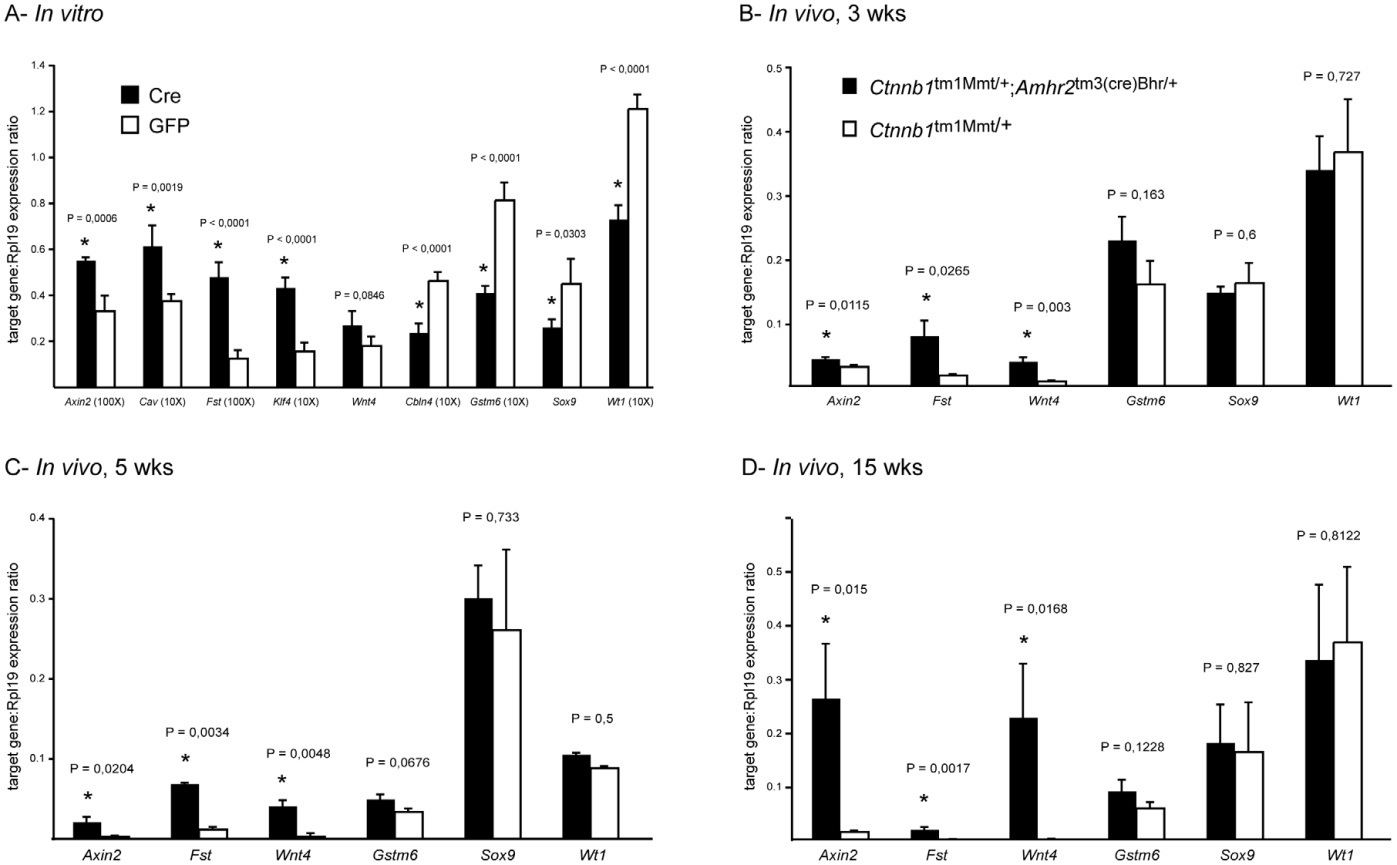
Expression and regulation of CTNNB1 target genes. (*A*) Quantitative RT-PCR analysis of the expression of the indicated genes in cultured *Ctnnb1* ^tm1Mmt/tm1Mmt^ Sertoli cells treated with the Ad-eGFP (control) or Ad-Cre adenovirus, n = 4 samples/treatment, performed in triplicate. (*B–D*) Quantitative RT-PCR analysis of the expression of the indicated genes in Sertoli cells isolated from testes of 3 week- (*B*), 5 week- (*C*) and 15 week- (*D*) old mice, n = 4 animals/genotype/time point performed in triplicate. All data were normalized to the housekeeping gene *Rpl19*, and are expressed as mean (columns) ± SEM (error bars). Relevant *P* values are indicated, and accompanied with an asterisk (*) when significant differences from controls (*P*<0.05) were detected.

We next sought to identify changes in Sertoli cell gene expression in response to sustained CTNNB1 signaling that could be responsible for the loss of SSC activity in *Ctnnb1* ^tm1Mmt/+^;*Amhr2* ^tm3(cre)Bhr/+^ mice. An *in vitro* approach using short-term primary Sertoli cell cultures was used in order to preferentially identify immediate CTNNB1 transcriptional targets in an isolated cell population. Sertoli cells from 3 week-old *Ctnnb1* ^tm1Mmt/tm1Mmt^ mice were placed in culture and infected with adenoviruses to induce the expression of either Cre recombinase (Ad-Cre, to express dominant-stable CTNNB1) or eGFP (Ad-eGFP, control) for 24 h. Then, each cell population was subjected to microarray analyses, followed by *in silico* analyses to identify groups of genes associated with specific signaling pathways or biological processes. A first group of genes induced by Ad-Cre (i.e., dominant-stable CTNNB1) consisted of *Ccnd1* (cyclin D1) and a number of cell cycle-associated genes with known interaction with cyclin D1 (Fig. S1, Table S1), suggesting that CTNNB1 could regulate Sertoli cell proliferation. Accordingly, abnormal Sertoli cell proliferation has been previously observed in *Ctnnb1* ^tm1Mmt/+^;*Amhr2* ^tm3(cre)Bhr/+^ mice, eventually leading to tumor development [20]–[22]. A second set of genes that were differentially expressed following Ad-Cre treatment were genes involved in sex differentiation, or that are differentially expressed between male and female genital ridges during embryonic development (Table S1). Specifically, genes normally associated with female gonadal development such as *Klf4* and *Fst* were upregulated in *Ctnnb1* ^tm1Mmt/tm1Mmt^ Sertoli cells treated with Ad-Cre, whereas male gonadal development genes such as *Sox9* and *Wt1* were downregulated. Microarray results were confirmed by real-time RT-PCR (Fig. 3A). Since *Wnt4* is known to be a CTNNB1 target gene in granulosa cells (equivalent to Sertoli cells in developmental origin) and its overexpression causes male-to-female sex reversal, we also analyzed *Wnt4* expression in our short-term in vitro system using real-time RT-PCR. As shown in Fig. 3A, there was a trend (*P*<0.1) that *Wnt4* was preferentially expressed in Ad-Cre-treated cells but no significant differences were detected.

To determine if genes identified in the *in vitro* approach are also differentially expressed *in vivo* during the course of germ cell loss, RT-PCR analysis was performed on Sertoli cells isolated from testes of 3, 5 and 15 week-old *Ctnnb1* ^tm1Mmt/+^;*Amhr2* ^tm3(cre)Bhr/+^ and control mice. As in the *in vitro* model, markers of female sex differentiation including *Wnt4* were upregulated in *Ctnnb1* ^tm1Mmt/+^;*Amhr2* ^tm3(cre)Bhr/+^ testes (Fig. 3B–3D). A clearly elevated level of *Wnt4* expression in Sertoli cells derived from mutant mice contrasts with our results of the in vitro assay (Fig. 3A), suggesting that *Wnt4* may not be an immediate target of CTNNB1 signaling but its expression may increase with time. On the other hand, markers of male sex differentiation were unchanged in *Ctnnb1* ^tm1Mmt/+^;*Amhr2* ^tm3(cre)Bhr/+^ testes, indicating an overall maintenance of the Sertoli/male phenotype in these mice; the reason for the discrepancy with the *in vitro* data is unclear. Nonetheless, these results collectively suggested that sustained activation of CTNNB1 signaling either drove cells committed to a Sertoli cell fate to express granulosa-cell related genes, or directed uncommitted somatic progenitor cells towards a granulosa cell-like state.

### WNT4 downregulates spermatogonial stem cell activity *in vitro*

**Figure 4:**
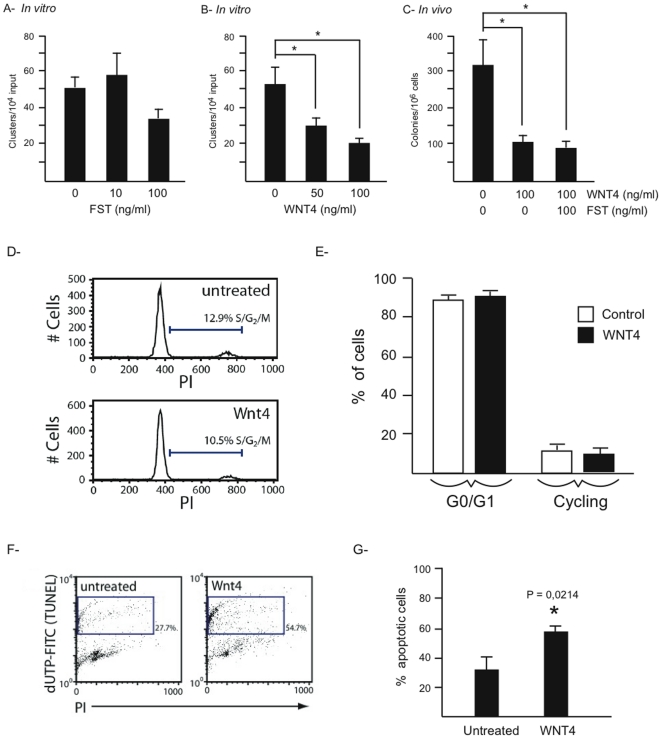
WNT4 downregulates SSC activity *in vitro*. (*A*, *B*) Germ cell cluster formation ability of cells treated with the indicated concentrations of FST (*A*) or WNT4 (*B*). (*C*) Colony numbers obtained after transplantation of germ cells treated with WNT4 or WNT4 and FST at the indicated concentrations. (*D*, *E*) Representative flow cytometric histograms (*D*) and analysis (*E*) showing the cell cycle profiles of cultured germ cells, with or without prior WNT4 treatment (100 ng/ml). (*F*, *G*) Representative scatter plots (*F*) and analysis (*G*) showing TUNEL assay results of germ cells following WNT4 treatment (100 ng/ml). All data are expressed as mean (columns) ± SEM (error bars). Significant differences from controls (*P*<0.05) are indicated with an asterisk (*) and accompanied by relevant *P* values.

As Sertoli cells regulate SSC activity via the secretion of paracrine factors, the above-mentioned gene expression data was carefully screened for the differential expression of genes encoding secreted molecules that could contribute to the progressive loss of SSC activity observed in *Ctnnb1* ^tm1Mmt/+^;*Amhr2* ^tm3(cre)Bhr/+^ mice. Although no such factors were found to be down-regulated by CTNNB1, both *Wnt4* and *Fst* were up-regulated. We therefore sought to determine if either could affect SSC activity *in vitro*. Testis cells from C57BL/6×*Gt(ROSA)26Sor* F_1_ hybrid mice were enriched for SSCs and cultured on feeder cells, resulting in the formation of undifferentiated spermatogonial aggregates (clusters). Clusters were then transferred onto Matrigel without feeder cells and treated with recombinant FST or WNT4 at different concentrations. After 4 days, the cluster-forming ability of the treated cells was assessed, as this is an in vitro indicator of SSC activity [18], [23]. Only WNT4 had a statistically significant effect, reducing cluster formation by 43% at a concentration of 50 ng/ml and by 61% at a concentration of 100 ng/ml (Fig. 4A, 4B). To confirm the effect of WNT4 on SSC activity, transplantation assays were also performed with WNT4-treated cells. Consistent with the in vitro assay results, WNT4 reduced SSC activity by 65% compared to controls (Fig. 4C). Cells treated with both WNT4 and FST did not reduce further colony numbers, suggesting that WNT4 is the principal factor affecting SSC activity and that there is no synergistic effect between the two factors.

**Figure 5:**
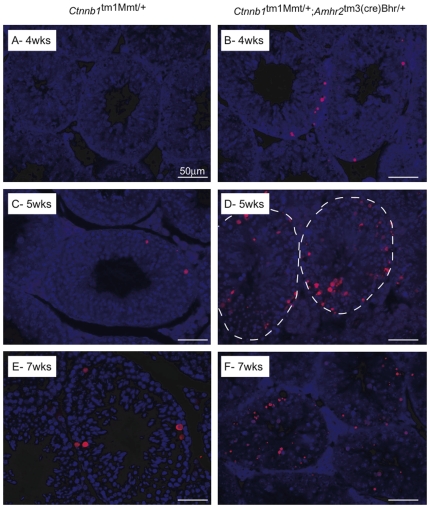
Increased germ cell apoptosis in *Ctnnb1* ^tm1Mmt/+^;*Amhr2* ^tm3(cre)Bhr/+^ testis. (*A–F*) TUNEL staining (red) in *Ctnnb1* ^tm1Mmt/+^;*Amhr2* ^tm3(cre)Bhr/+^ testes (*B*, *D*, *F*) compared with *Ctnnb1^tm1Mmt/+^* controls (*A*, *C*, *E*) at different ages. Counterstain = DAPI (blue). For clarity, seminiferous tubules are circumscribed with a dotted white line in panel *D*.

To determine if the loss of SSC activity *in vitro* in response to WNT4 could be attributed to reduced proliferation, the cell cycle profile of the WNT4-treated cluster cells was examined by FACS analysis following propidium iodide incorporation. Results showed statistically indistinguishable proportions of cells in the proliferative phase (S/G_2_/M) of the cell cycle between WNT4-treated and control cells, indicating that WNT4 did not affect proliferation (Fig. 4D, 4E). We next examined the effects of WNT4 on apoptosis in cluster cells. Twice as many TUNEL-positive cells were detected in the group treated with WNT4 relative to the control (54.7% vs 27.7%) (Fig. 4F, 4G), indicating that WNT4 hinders the survival of germ cells *in vitro*. Accordingly, TUNEL analyses of testes from 5 week-old *Ctnnb1* ^tm1Mmt/+^;*Amhr2* ^tm3(cre)Bhr/+^ mice (an age at which WNT4 is already overexpressed, Fig. 3C) showed a precipitous increase in germ cell apoptosis, including germ cells situated at the periphery of the tubules, where spermatogonial populations are located (Fig. 5). The timing of increased apoptosis roughly coincided with the onset of testicular atrophy and germ cell loss in the *Ctnnb1* ^tm1Mmt/+^;*Amhr2* ^tm3(cre)Bhr/+^ model [19], and is similar to observations made by Tanwar et al. [20]. Together with a significant reduction in SSC numbers in 5-week-old mutant mouse testes (Fig. 1), this result suggests that WNT4 may also induce SSC apoptosis *in vivo*.

### WNT4 signals via distinct pathways in SSCs and Sertoli cells

**Figure 6:**
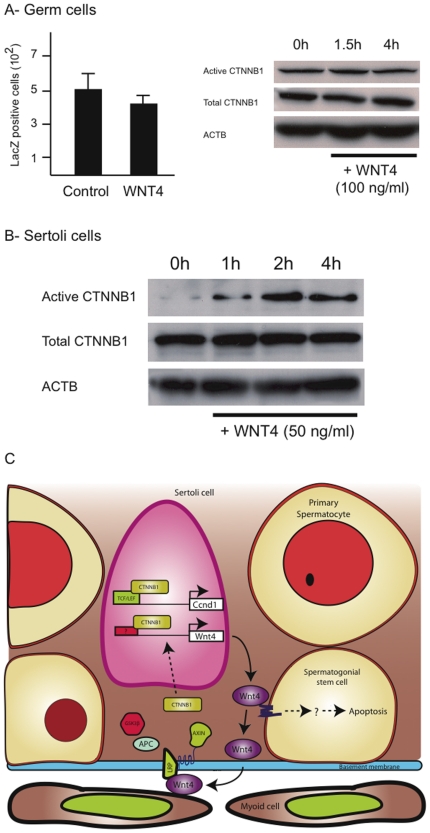
WNT4 acts through canonical and noncanonical pathways in different testicular cell types. (*A*) Left panel: LacZ-positive cell numbers in cultured germ cells from TCF/Lef-lacZ transgenic mice treated or not beforehand with WNT4 (100 ng/ml). Data is expressed as mean (columns) ± SEM (error bars). Right panel: Timecourse immunoblot analyses of cultured germ cells treated with WNT4 (100 ng/ml). ACTB was used as a loading control. (*B*) Timecourse immunoblot analyses of cultured Sertoli cells treated with WNT4 (50 ng/ml). ACTB was used as a loading control. (*C*) Experimental model illustrating WNT4/CTNNB1 signaling mechanisms in Sertoli cells and spermatogonial stem cells.

To study the signaling mechanism through which WNT4 acts, SSCs were isolated from a TCF/Lef-LacZ transgenic mouse strain [24]. Cells from these mice respond to canonical WNT signals by an increase in lacZ expression [18], [24]. SSC culture was established using germ cells of TCF/Lef-LacZ mice. Cluster cells generated were then transferred to the feeder-free condition and exposed to WNT4. The effect on SSC activity was assessed using the in vitro assay, as in Fig. 4. No differences were observed in the numbers of LacZ-expressing cells between control and WNT4-treated cells (Fig. 6A). In a second experiment, cluster cells were treated with WNT4, and levels of active (i.e., dephosphorylated) CTNNB1 were determined by western blot. Consistent with the results of the first experiment, WNT4 had no effect on active CTNNB1 (Fig. 6A). These results indicate that WNT4 does not activate the canonical pathway in spermatogonia.

To determine if WNT4 can also act in an autocrine manner in Sertoli cells, primary cultured Sertoli cells were treated with WNT4 and levels of active CTNNB1 were determined by western blot. Increased active CTNNB1 was detected as early as 1 h after exposure to WNT4 (Fig. 6B), indicating that WNT4 can act directly on Sertoli cells through the canonical pathway. As *Wnt4* is a CTNNB1 target gene in Sertoli cells (Fig. 3), this result suggests a potential positive feedback loop through which WNT4 and CTNNB1 mutually increase their expression (Fig. 6C).

### WNT4 acts downstream of CTNNB1 to cause germ cell loss *in vivo*

**Figure 7:**
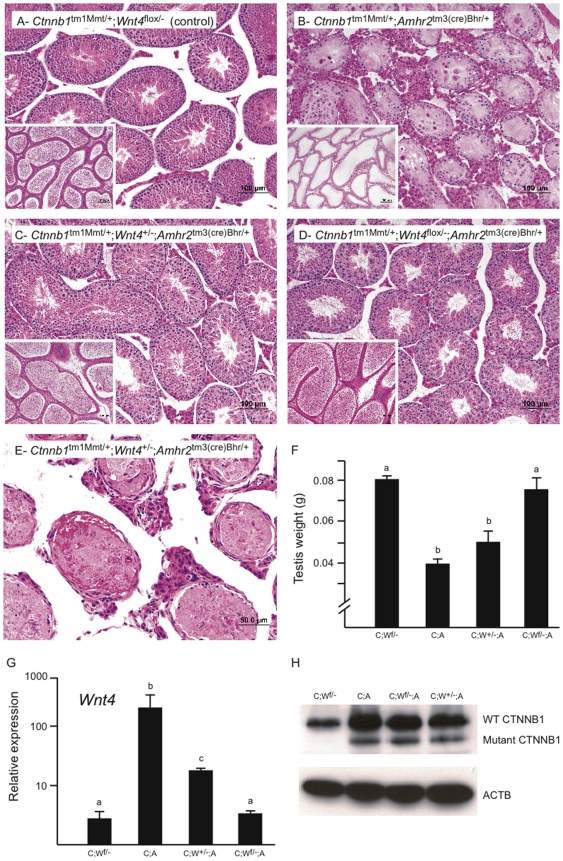
WNT4 acts downstream of CTNNB1 to cause germ cell loss *in vivo*. (*A*–*D*) Photomicrographs of testes from 8 week-old animals of the indicated genotypes. Insets show sections of epididymides from the corresponding animals. (*E*) Photomicrograph of a testis of the indicated genotype showing the severe testicular degeneration, coagulation necrosis and intratubular hemorrhage phenotypes described in the text. (*F*) Testis weights from 8 week-old animals of the indicated genotypes, n = 4 animals/genotype. C;W^f/−^: *Ctnnb1* ^tm1Mmt/+^;*Wnt4* ^flox/−^ (control), C;A: *Ctnnb1* ^tm1Mmt/+^;*Amhr2* ^tm3(cre)Bhr/+^, C;W^+/−^;A: *Ctnnb1* ^tm1Mmt/+^;*Wnt4* ^+/−^;*Amhr2* ^tm3(cre)Bhr/+^, C;W^f/−^;A: *Ctnnb1* ^tm1Mmt/+^;*Wnt4* ^flox/−^;*Amhr2* ^tm3(cre)Bhr/+^. (*G*) *Wnt4* mRNA levels in the mice described in panel F. Note the logarithmic scale on the Y axis. (*H*) CTNNB1 immunoblot analyses of testes from 8 week-old animals of the indicate genotypes. The lower band corresponds to the dominant-stable CTNNB1 mutant protein produced by the recombined *Ctnnb1* ^tm1Mmt/+^ allele. ACTB was used as a loading control. Animals showing the severe degenerative phenotype described in the text and shown in panel *E* were excluded from the data analyses shown in panels *F*–*H*. All data are expressed as mean (columns) ± SEM (error bars). Groups labeled with different letters (a, b, c) were significantly different (*P*<0.05).

Based on the results described in Figs. 4 and 5, we next hypothesized that WNT4 is the major effector downstream of CTNNB1 responsible for SSC and germ cell loss in the *Ctnnb1* ^tm1Mmt/+^;*Amhr2* ^tm3(cre)Bhr/+^ model. To test this, a floxed *Wnt4* allele was introduced into the *Ctnnb1* ^tm1Mmt/+^;*Amhr2* ^tm3(cre)Bhr/+^ background so as to conditionally inactivate *Wnt4* in the same cells in which CTNNB1 signaling had been constitutively activated. The resulting *Ctnnb1* ^tm1Mmt/+^;*Wnt4* ^flox/−^;*Amhr2* ^tm3(cre)Bhr/+^ male mice were fertile at least up to the age of 7 months, when the mating experiments were terminated (not shown). *Ctnnb1* ^tm1Mmt/+^;*Wnt4* ^flox/−^;*Amhr2* ^tm3(cre)Bhr/+^ testis weights at 8 wks were significantly higher than those of *Ctnnb1* ^tm1Mmt/+^;*Amhr2* ^tm3(cre)Bhr/+^ mice, and indistinguishable from *Ctnnb1* ^tm1Mmt/+^;*Wnt4* ^flox/−^ controls (Fig. 7F). Histopathological analysis of *Ctnnb1* ^tm1Mmt/+^;*Wnt4* ^flox/−^;*Amhr2* ^tm3(cre)Bhr/+^ testes revealed qualitatively normal spermatogenesis in the vast majority of seminiferous tubules, along with abundant spermatotozoa in the epididymides in animals up to 5 months of age (Fig. 7D and not shown). Real-time RT-PCR analysis confirmed a near-complete loss of *Wnt4* expression in *Ctnnb1* ^tm1Mmt/+^;*Wnt4* ^flox/−^;*Amhr2* ^tm3(cre)Bhr/+^ testes (Fig. 7G), whereas the dominant-stable CTNNB1 mutant protein was expressed at levels comparable to *Ctnnb1* ^tm1Mmt/+^;*Amhr2* ^tm3(cre)Bhr/+^ controls (Fig. 7H). *Fst* expression was also downregulated in *Ctnnb1* ^tm1Mmt/+^;*Wnt4* ^flox/−^;*Amhr2* ^tm3(cre)Bhr/+^ compare to *Ctnnb1* ^tm1Mmt/+^;*Amhr2* ^tm3(cre)Bhr/+^ testes (Fig. S2), suggesting that even though FST is not involved in the regulation of SSC activity, *Fst* is a downstream target of WNT4. Interestingly, control mice bearing a single functional *Wnt4* allele (*Ctnnb1* ^tm1Mmt/+^;*Wnt4* ^+/−^;*Amhr2* ^tm3(cre)Bhr/+^) had testicular *Wnt4* and *Fst* expression levels and testis weights intermediate between the *Ctnnb1* ^tm1Mmt/+^;*Amhr2* ^tm3(cre)Bhr/+^ and *Ctnnb1* ^tm1Mmt/+^;*Wnt4* ^flox/−^;*Amhr2* ^tm3(cre)Bhr/+^ groups, and this was accompanied by a partial rescue of spermatogenesis, as evidenced by a delayed degeneration of the tubules and the presence of spermatozoa in the epididymides (Fig. 7C, 7F, 7G). Unexpectedly, we also observed that some (∼30%) animals of both the *Ctnnb1* ^tm1Mmt/+^;*Wnt4* ^flox/−^;*Amhr2* ^tm3(cre)Bhr/+^ and *Ctnnb1* ^tm1Mmt/+^;*Wnt4* ^+/−^;*Amhr2* ^tm3(cre)Bhr/+^ genotypes had a much more severe phenotype. Testes of these animals were very small (<3 mg) and showed degeneration and coagulation necrosis of the seminiferous epithelium, intratubular hemorrhages and dystrophic mineralization (Fig. 7E and not shown). Although this severe phenotype remained unexplained, a breakdown of the blood-testis barrier was apparently involved. Nonetheless, the rescue of spermatogenesis and fertility observed in most *Ctnnb1* ^tm1Mmt/+^;*Wnt4* ^flox/−^;*Amhr2* ^tm3(cre)Bhr/+^ mice indicates that WNT4 is the major effector downstream of CTNNB1 that is responsible for testicular degeneration, SSC and germ cell loss and sterility in *Ctnnb1* ^tm1Mmt/+^;*Amhr2* ^tm3(cre)Bhr/+^ mice.

## Discussion

The SSCs are the adult stem cell population that sits atop the male germ cell developmental hierarchy. The proper regulation of SSC activity is therefore critical for the initiation and maintenance of spermatogenesis. SSC survival and activity is dependent on a highly specialized microenvironment, the SSC niche, which provides the paracrine factors required to nurture SSCs and regulate their cell division patterns to maintain spermatogenesis. Various mutant mice have been reported in which spermatogenesis is disrupted and SSC activity declines. Phenotypes of some of these mutants are known to arise from defects in Sertoli cells, an important element of the SSC niche. Two examples of such mutants are Steel (Sl) and ETV5-knockout mice. In Sl mice, mutations occur in a Sertoli cell-derived growth factor, KITL, and affect the survival and function of differentiating spermatogonia, rather than SSCs, in postnatal mice and those of primordial germ cells in embryos, resulting in the loss of spermatogenesis [25], [26]. Although available data indicate that the SSC population size is diminished in Sl mouse testes, SSCs remaining are capable of accomplishing complete spermatogenesis when exposed to wild-type Sertoli cells [27], [28]. In the case of ETV5-knockout mice, spermatogenesis takes place during the first cycle after birth but ceases with time due to gradual depletion of SSCs. ETV5-null mutation seems to affect both SSCs and Sertoli cells, and the mechanism of SSC depletion has not been identified definitively [29]–[30].

Here, we report that activation of CTNNB1 signaling specifically in Sertoli cells results in a defective testicular somatic environment that negatively regulates SSC activity. Our results indicate that *Ctnnb1* ^tm1Mmt/+^;*Amhr2* ^tm3(cre)Bhr/+^ testes lose their endogenous SSC activity over time and are unable to support SSCs upon transplantation. These findings suggested an alteration of the paracrine signals emanating from Sertoli cells, leading to a functional breakdown of the SSC niche and resultant loss of SSC activity. Our subsequent investigations identified WNT4 as a key paracrine factor that acts downstream of CTNNB1 to downregulate SSC activity. Not only could WNT4 reduce SSC activity and increase germ cell apoptosis *in vitro*, but conditional inactivation of *Wnt4* in the *Ctnnb1* ^tm1Mmt/+^;*Amhr2* ^tm3(cre)Bhr/+^ model rescued spermatogenesis and restored fertility. We therefore conclude that, rather surprisingly, WNT4 appears to be the major factor downstream of CTNNB1 that is responsible for the loss of SSC activity, the inability to support donor SSCs, and the collapse of spermatogenesis that occurs in *Ctnnb1* ^tm1Mmt/+^;*Amhr2* ^tm3(cre)Bhr/+^ mice. It was previously suggested that GDNF might play a role in the induction of germ cell apoptosis in *Ctnnb1* ^tm1Mmt/+^;*Amhr2* ^tm3(cre)Bhr/+^ testes [20]. Contrary to Tanwar et al., we did not find increased expression of *Gdnf* in the *Ctnnb1* ^tm1Mmt/+^;*Amhr2* ^tm3(cre)Bhr/+^ model (Fig. S2), although we examined mRNA levels whereas Tanwar et al. studied protein expression, and therefore the stabilization of GDNF protein in *Ctnnb1* ^tm1Mmt/+^;*Amhr2* ^tm3(cre)Bhr/+^ testes could explain this discrepancy. Regardless, our results suggest that WNT4 is the primary factor involved in germ cells apoptosis, but do not exclude that GDNF might also play a minor role.

The identification of WNT4 as a negative regulator of SSC activity may provide insight into the pathogenesis of male infertility. The *Ctnnb1* ^tm1Mmt/+^;*Amhr2* ^tm3(cre)Bhr/+^ model closely mimics certain forms of Sertoli Cell Only Syndrome (SCOS), being characterized by germ cell loss, normal virilization, and a mosaic pattern of Sertoli cell differentiation in the adult testis with the expression of markers of immature Sertoli cells [19], [22]. The pathogenesis of SCOS is poorly understood at the genetic level, although microdeletions of the Y chromosome are involved in some cases [31], [32]. Our results suggest that any post-developmental event resulting in the inappropriate activation of the WNT4/CTNNB1 signaling loop in Sertoli cells could lead to SSC loss, with subsequent loss of all germ cells. As WNT4 is a secreted molecule that can act in a paracrine manner and can amplify its own signal (Fig. 6C), localized inappropriate activation of this pathway could conceivably spread to affect the entire organ. Whether or not such a mechanism is relevant to the etiology of SCOS in men has not been studied to our knowledge.

Importantly, our findings that WNT4/CTNNB1 signaling in Sertoli cells results in the expression of granulosa cell genes and downregulates SSC activity may provide new insight into mechanisms of sex determination and gonadal development. WNT4/CTNNB1 signaling has well-established roles in female sex determination, as WNT4 insufficiency during embryogenesis results in partial female-to-male sex reversal [33]–[35]. Conversely, WNT4/CTNNB1 overexpression during embryonic development can cause male-to-female sex reversal, the extent of which apparently depending both on species and the amount of WNT4 expression [36]–[39]. In this study, we demonstrate that constitutive WNT4/CTNNB1 signaling in cells already committed to the Sertoli cell fate results in the expression of genes normally expressed during female sex differentiation and a loss of Sertoli cell differentiation and function (see also [19]). These results are in agreement with the recent view that gonadal somatic cell commitment to either the granulosa cell or Sertoli cell fate is not “stable”, and can be partially or completely reversed by alterations in specific signaling processes [40]. Perhaps the most dramatic example of this concept is the recent report that postnatal deletion of *Foxl2* results in the reprogramming of granulosa cells into a Sertoli-like cell lineage [41].

Interestingly, WNT5a has recently been shown to promote SSC activity via non-canonical pathways [18]. As WNT4 also appears to signal via non-canonical mechanisms in SSCs, it remains to be determined how each molecule acts to generate opposite biological effects via a similar mechanism in a single cell type. One possibility is that they have distinct receptor complexes, the activation of which triggers distinct (but partially overlapping) signaling cascades [42]. This idea is supported by the observation that WNT5a appears to actively repress CTNNB1 signaling in SSCs [18], which we did not observe in the case of WNT4.

We believe that it is unlikely that WNT4 plays a physiological role in the regulation of SSC activity in the adult testis. Indeed, we found *Wnt4* mRNA to be barely detectable or undetectable in normal testes using PCR-based methods (Fig. 3, Fig. S3), whereas the expression of most other *Wnt* genes (including *Wnt5a*) was readily detectable (Fig. S3). Furthermore, *Wnt4* ^flox/−^;*Amhr2* ^tm3(cre)Bhr/+^ mice are fertile and have qualitatively normal spermatogenesis (unpublished observations). Our data therefore seem to indicate that the WNT4/CTNNB1 pathway is not a physiological regulator of spermatogenesis. It should further be noted that Wnt1, a potent activator of the WNT/CTNNB1 pathway, is robustly produced by spermatids, which represent over 70% of cells in the seminiferous epithelium [43], [44]. This implies that Sertoli cells are constantly exposed to the activator of WNT/CTNNB1 signaling, yet the pathway is not normally active in these cells [18], [19]. Collectively, these results therefore argue that the canonical WNT signaling pathway must be suppressed (presumably by repressors of WNT signaling such as SFRPs, DDKs or NKDs) in Sertoli cells to maintain the functional integrity of SSC niche, and thus the forced activation of this pathway has catastrophic consequences for SSCs, spermatogenesis, and male fertility.

In summary, we have identified a WNT4/CTNNB1 signaling loop in Sertoli cells that acts in a paracrine manner to downregulate SSC activity. The results of this study may provide important insight into gonadal development and into the etiology of certain male germ cell loss pathologies.

## Materials and Methods

### Transgenic mouse strains

*Gt(ROSA)26Sor;Ctnnb1* ^tm1Mmt/+^;*Amhr2* ^tm3(cre)Bhr/+^, *Gt(ROSA)26Sor;Ctnnb1* ^tm1Mmt/+^ and *Ctnnb1* ^tm1Mmt/+^;*Amhr2* ^tm3(cre)Bhr/+^ mice were derived from previously-described *Amhr2^tm3(cre)Bhr/+^* [45], *Ctnnb1^tm1Mmt^* [46] and *B6.129S7-Gt(ROSA)26Sor/J* (Jackson Laboratory, Bar Harbor, ME, stock number 002192) parental strains by selective breeding. Genotypes were determined by PCR as described [46], [47]. TCF/Lef-LacZ transgenic mice were a kind gift from Dr Daniel Dufort (McGill University) [24]. *Wnt4* null and floxed alleles were developed in our laboratory and genotype analyses were done as previously described [48]. All animal procedures were approved by the Comité d'Éthique de l'Utilisation des Animaux of the Université de Montréal (permit number 11-RECH-1320 and 11-RECH-1488) and were conform to the USPHS Policy on Humane Care and Use of Laboratory Animals.

### Germ cell transplantations

Donor mice were *Gt(ROSA)26Sor*, *Gt(ROSA)26Sor;Ctnnb1* ^tm1Mmt/+^ and *Gt(ROSA)26Sor;Ctnnb1* ^tm1Mmt/+^;*Amhr2* ^tm3(cre)Bhr/+^. *Gt(ROSA)26Sor* mice express the *Escherichia coli lacZ* transgene in virtually all cell types, including all types of postnatal male germ cells, allowing the discrimination of donor cells from recipient cells after transplantation *in vivo* and from feeder cells in culture [49], [50]. Donor cell suspensions were prepared from testes from 5- or 17 week-old mice using a previously-described two-step enzymatic digestion protocol [51], [52], except 1 mg/ml of collagenase I, 1 mg/ml collagenase IV, 1 mg/ml hyaluronidase and 1 mg/ml DNase I (Sigma, St. Louis, MO) were used in the first step. Transplant recipients were *Ctnnb1* ^tm1Mmt/+^;*Amhr2* ^tm3(cre)Bhr/+^, *Ctnnb1* ^tm1Mmt/+^ and wild-type 129/SvEv×B6 F_1_ hybrid mice. To deplete endogenous spermatogenesis, recipient mice were treated with busulfan (50 mg/kg, i.p.) at 6 weeks of age [51], [52]. Six weeks later, donor SSC-enriched cells were resuspended at 1.0–1.2×10^6^ cells/ml, and injected into recipient seminiferous tubules through the efferent duct as previously described [51], [52]. For SSC quantification, recipient testes were harvested at 1, 5 or 8 weeks after transplantation, and stained with X-gal to visually count the colonies of donor-derived spermatogenesis as previously described [53].

### Sertoli cell culture, adenoviral infection and microarray analyses

Sertoli cells were isolated from 3 week-old *Ctnnb1* ^tm1Mmt/tm1Mmt^ animals as previously described [39]. After 2 days of culture in DMEM containing 10% fetal calf serum, cells were infected with adenoviruses to express either eGFP or Cre recombinase for 24 h in serum-free medium, and subsequently harvested for RNA extraction as described below. Ad-Cre and Ad-eGFP viruses were obtained from the Baylor College of Medicine Vector Development Laboratory (Houston, TX, USA). Preliminary experiments demonstrated that an infection efficiency of nearly 100% could be obtained at an MOI of ∼50 (as determined by analysis of fluorescent signal in Ad-eGFP-infected cells), and that recombination of the floxed *Ctnnb1* alleles was complete 12 h after the infection with Ad-Cre (as determined by PCR-based genotype analyses) (not shown). The recombined *Ctnnb1* ^tm1Mmt^ allele encodes a truncated, “dominant-stable” CTNNB1 mutant protein that, while still fully functional, is resistant to degradation and therefore accumulates to abnormally high levels in the cell, thereby altering the expression of CTNNB1 transcriptional target genes [46]. Microarray analyses were done using triplicate RNA samples from each adenoviral treatment, and using MouseRef-6 v.2.0 expression BeadChips technology (Illumina, San Diego, CA, USA). All steps of RNA quality control, probe synthesis, hybridization, washing, and array scanning were done by McGill University and the Génome Québec Innovation Center (Montréal, Qc, Canada). Microarray data were analyzed using FlexArray 1.3 software (Génome Québec). Ad-Cre and Ad-eGFP data were processed using t-test, the EB (Wright and Simon) algorithm and P-value calculation. A P-value threshold of 0.05 and 1.5-fold change cut-off values were used for identification of differentially expressed genes. All array data are MIAME compliant and the raw data were deposited in the MIAME compliant database GEO, with accession number GSE28402. Array data were also analyzed with Pathway Studio (Ariadne, Rockville, MD) to identify functional interactions between genes.

### Real-time RT-PCR

Gene expression analyses were done by real-time RT-PCR on Sertoli cells isolated from from 3, 5 or 15 week-old mice that were either cultured or freshly-isolated as described above. Briefly, RNA samples were purified with the RNeasy mini kit (Qiagen, Valencia, CA). RT-PCR reactions were formulated using the Superscript III Platinum two-step qRT-PCR kit with SYBR green (Invitrogen, Burlington, ON, Canada) according to the manufacturer's instructions and using the oligonucleotide primer pairs listed in Table S2. Thermal cycling and data capture were performed using a Rotor-Gene RG-3000 apparatus (Corbett Research, Mortlake NSW 2137, Australia) with the manufacturer's recommended conditions. Relative gene expression was calculated using Rotor-Gene 6.0 software (Corbett Research) by comparing amplification curves to a series of standard curves obtained by amplification of serial dilutions of an *Rpl19* cDNA fragment. All data were subsequently normalized by dividing expression levels of individual genes by corresponding *Rpl19* values. The *Wnt4* RT-PCR analyses described in Fig. 6 were conducted as described above, except RNA was harvested from whole testes from 8 week-old mice of the indicated genotypes.

### Spermatogonial stem cell culture and cluster analysis

SSCs were enriched from C57BL/6×*Gt(ROSA)26Sor* F1 hybrid mouse testes and cultured as previously described [23]. SSCs proliferate in culture as distinct germ cell accumulations termed “clusters” [54], [55]. All *in vitro* experiments were conducted using established cluster cultures (i.e. >5 passages; 30 days). To avoid potential confounding effects associated with the presence of STO feeder cells during experimental treatments, clusters were removed from feeder cells by gentle pipetting, dissociated into single cells with 0.05% trypsin-EDTA and seeded (4–9×10^4^ cells/cm^2^) on culture dishes treated with Matrigel (BD Biosciences, Mississauga, ON, Canada). To examine effect of Follistatin (FST) or WNT4 on cultured cells, recombinant FST and/or WNT4 (R&D Systems) were added at the indicated concentrations (Fig. 4A, B) at the time of seeding. Cultures were maintained for 4 days and subsequently trypsinized and seeded back onto feeder cells (1∶1 split) under our standard condition to induce cluster growth. Clusters were visually counted following fixation and X-gal staining as previously described [18]. The cluster assay was performed in triplicate. To further examine SSC activity by spermatogonial transplantation, cluster cells were cultured on Matrigel and treated as described above, collected using 0.25% trypsin-EDTA, resuspended at 1.8–3.2×10^6^ cells/ml and transplanted into recipient testes. Spermatogonial transplantation was performed in duplicate, and colonies were counted in n>11 total recipient testes for each treatment group (Fig. 4C).

### *In vitro* proliferation, apoptosis and TCF/Lef-LacZ reporter transgene analyses

To evaluate the effects of WNT4 on germ cell proliferation, C57BL/6×*Gt(ROSA)26Sor* F_1_ cluster cells were seeded onto Matrigel at 10–12×10^4^ cells/cm^2^ as described above, with or without recombinant WNT4 (100 ng/ml) for four days. Cells were then trypsinized and fixed in 70% EtOH for 30 min or overnight. Propidium iodide (40 µg/ml) and RNAse (100 µg/ml) were added and cells incubated at 37°C for 30 min. Cell cycle profiles were obtained using a FACScan apparatus, with 5,000–10,000 events collected per sample. The percentage of cells in each phase of the cell cycle was determined using FloJo Flow Cytometry Analysis Software (TreeStar, Ahland, OR). The experiment was performed in triplicate. To evaluate the effects of WNT4 on apoptosis, germ cells were cultured and treated as described above for the cell cycle analyses. Cells actively undergoing apoptosis were identified by terminal deoxynucleotidyl transferase dUTP nick-end labeling (TUNEL) using the APO-Direct Apoptosis Detection Kit (BD Biosciences) according to the manufacturer's instructions, followed by FACS analysis (5,000–10,000 events recorded per sample). Results from four independent experiments are shown in Fig. 4G. To quantify the effects of WNT4 on WNT/CTNNB1 signaling, TCF/LEF-LacZ reporter mice were used, which have copies of the LacZ transgene under control of TCF/LEF responsive elements thereby allowing faithful monitoring of WNT/CTNNB1 activity. Germ cell clusters cultured from TCF/Lef-LacZ testes were seeded onto Matrigel-coated wells at 4–6×10^4^ cells/cm^2^ and cultured and treated as described above. On day 4, cells were trypsinized and reacted with X-gal overnight. Positive-staining cells (indicative of CTNNB1-TCF/Lef signaling activity) were then counted using a hemocytometer. The experiment was performed in triplicate.

### Immunoblot analyses

Sertoli cells from testes of 3 week-old C57BL/6 (B6) animals were isolated as described above. After 2 days in culture in DMEM containing 10% fetal calf serum, cells were put in serum-free medium for 24 hours. Recombinant WNT4 was added at a concentration of 50 ng/ml to the medium for 1, 2 or 4 hours. Cluster cells were cultured in serum-free medium under feeder-free conditions on Matrigel for 16 hours, and WNT4 (100 ng/ml) was added for 90 mins or 4 hours. Cell protein extracts were prepared using M-Per solution (Pierce, Rockford, IL) as directed by the manufacturer. The recovered supernatant was stored at −80°C until electrophoretic analyses were performed. Protein concentrations were determined by the Bradford method (Bio-Rad protein assay, Bio-Rad Laboratories, Hercules, CA). Samples were resolved by one-dimensional SDS-PAGE (12.5% acrylamide) under reducing conditions and electrophoretically transferred to a PVDF membrane (GE Amersham, Piscataway, NJ). The membrane was sequentially probed with antibodies against active CTNNB1 (Millipore, Billerica, MA, catalog #05-665), total CTNNB1 (Cell Signaling, Danvers, MA, catalog #9587) and ACTA (Santa Cruz Biotechnology, Santa Cruz, CA, catalog #sc-8432) diluted in 5% BSA. Following incubation with horseradish peroxidase-conjugated secondary rabbit anti-mouse antibody (GE Amersham), the protein bands were visualized by chemiluminescence using the ECL Plus Western Blotting Detection Reagents (GE Amersham) and High Performance Chemiluminescence film (GE Amersham). The immunoblots shown in Fig. 6 were done as described above, except proteins were isolated from testes of 8 week-old animals of the indicated genotypes.

### TUNEL analysis

Terminal deoxynucleotidyl transferase dUTP nick-end labeling assay was performed on Bouins-fixed, paraffin-embedded, 7-µm testis sections using the In Situ Cell Death Detection Kit, TMR red (Roche, Laval, Qc, Canada) as directed by the manufacturer. Slides were mounted using VectaShield with 4′,6-diamidino-2-phenylindole (DAPI) (Vector Labs, Burlingame, CA).

### Statistical Analyses

Statistical analyses for multiple comparisons were done using ANOVA followed by the Tukey post hoc test. Student *t*-test was used for two-group comparisons. Means were considered significantly different when *P*<0.05.

## Supporting Information

Figure S1 **Cyclin D1 (CCND1) and a network of CCND1-interacting genes are targets of CTNNB1 in Sertoli cells.** Known interactions between CCND1 and other genes that are up-regulated by CTNNB1 in cultured Sertoli cells are illustrated. The image was generated using Pathway Studio software.
(TIF)

Figure S2 ***Fst*** **and** ***Gdnf*** **expression in transgenic mouse testes.** (A) *Fst* mRNA levels from 8 week-old animals of the indicated genotypes, n = 4 animals/genotype. C;W^f/−^: *Ctnnb1* ^tm1Mmt/+^;*Wnt4* ^flox/−^ (control), C;A: *Ctnnb1* ^tm1Mmt/+^;*Amhr2* ^tm3(cre)Bhr/+^, C;W^+/−^;A: *Ctnnb1* ^tm1Mmt/+^;*Wnt4* ^+/−^;*Amhr2* ^tm3(cre)Bhr/+^, C;W^f/−^;A: *Ctnnb1* ^tm1Mmt/+^;*Wnt4* ^flox/−^;*Amhr2* ^tm3(cre)Bhr/+^. (B) *Gdnf* mRNA levels in the mice described in A. *Gdnf* expression was also evaluated in testes from 4 day-old wild-type mice, so as so confirm its physiological decrease during postnatal development. Data are expressed as mean (columns) ± SEM (error bars). Groups labeled with different letters (a, b, c) were significantly different (*P*<0.05).
(TIF)

Figure S3 **Analysis of the expression of** ***Wnt*** **and** ***Fzd*** **family members in the adult mouse testis.** The expression of each gene was assessed by semi-quantitative RT-PCR analysis following the indicated numbers of PCR cycles. PCR products were separated by agarose gel electrophoresis, stained with ethidium bromide, and photographed under UV light. As shown, some degree of expression of all *Wnt* and *Fzd* genes was detected, with the exceptions of *Fzd2*, *Wnt4*, *Wnt7b*, *Wnt8a*, *Wnt10a* and *Wnt16*.
(TIF)

Table S1 **Gene regulation in Sertoli cells in response to dominant-stable CTNNB1.** Microarray analyses of cultured Sertoli cells from 3 week-old *Ctnnb1* ^tm1Mmt/tm1Mmt^ mice infected for 24 h with adenoviruses to induce the expression of either Cre recombinase (Ad-Cre, to express dominant-stable CTNNB1) or eGFP (Ad-eGFP, control).
(DOC)

Table S2 **Oligonucleotide primer sequences.** List of primer sequences used for gene expression analyses by real-time RT-PCR.
(DOC)
