# Supplementary figures and images for: CTNNB1 Signaling in Sertoli Cells Downregulates Spermatogonial Stem Cell Activity via WNT4
Source: PLoS One. 2012 Jan 12;7(1):e29764. doi: 10.1371/journal.pone.0029764 (PMC3257228; doi:10.1371/journal.pone.0029764)

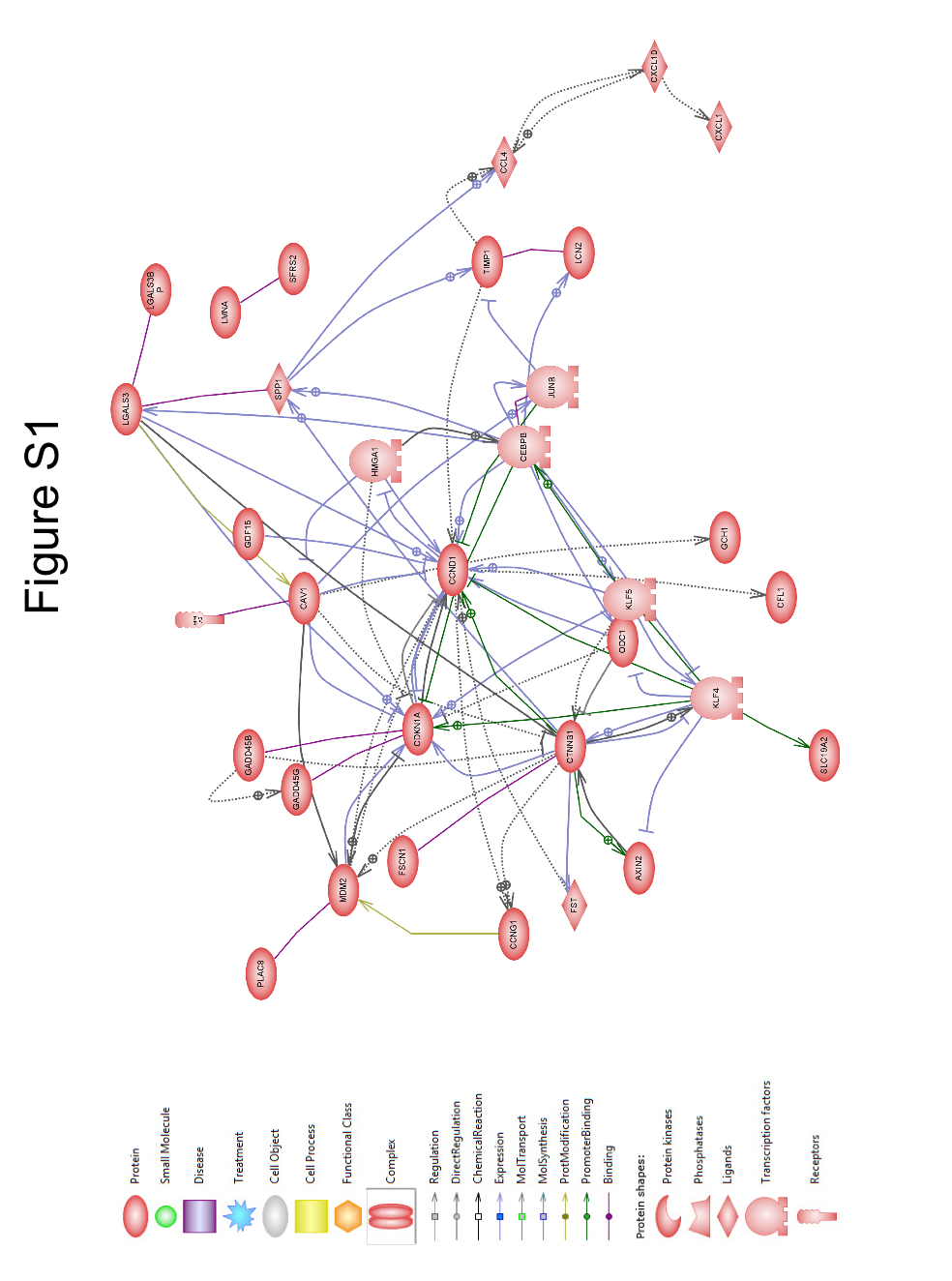

Supplement: Figure S1 — Cyclin D1 (CCND1) and a network of CCND1-interacting genes are targets of CTNNB1 in Sertoli cells. Known interactions between CCND1 and other genes that are up-regulated by CTNNB1 in cultured Sertoli cells are illustrated. The image was generated using Pathway Studio software. (TIF) [file pone.0029764.s001.tif]

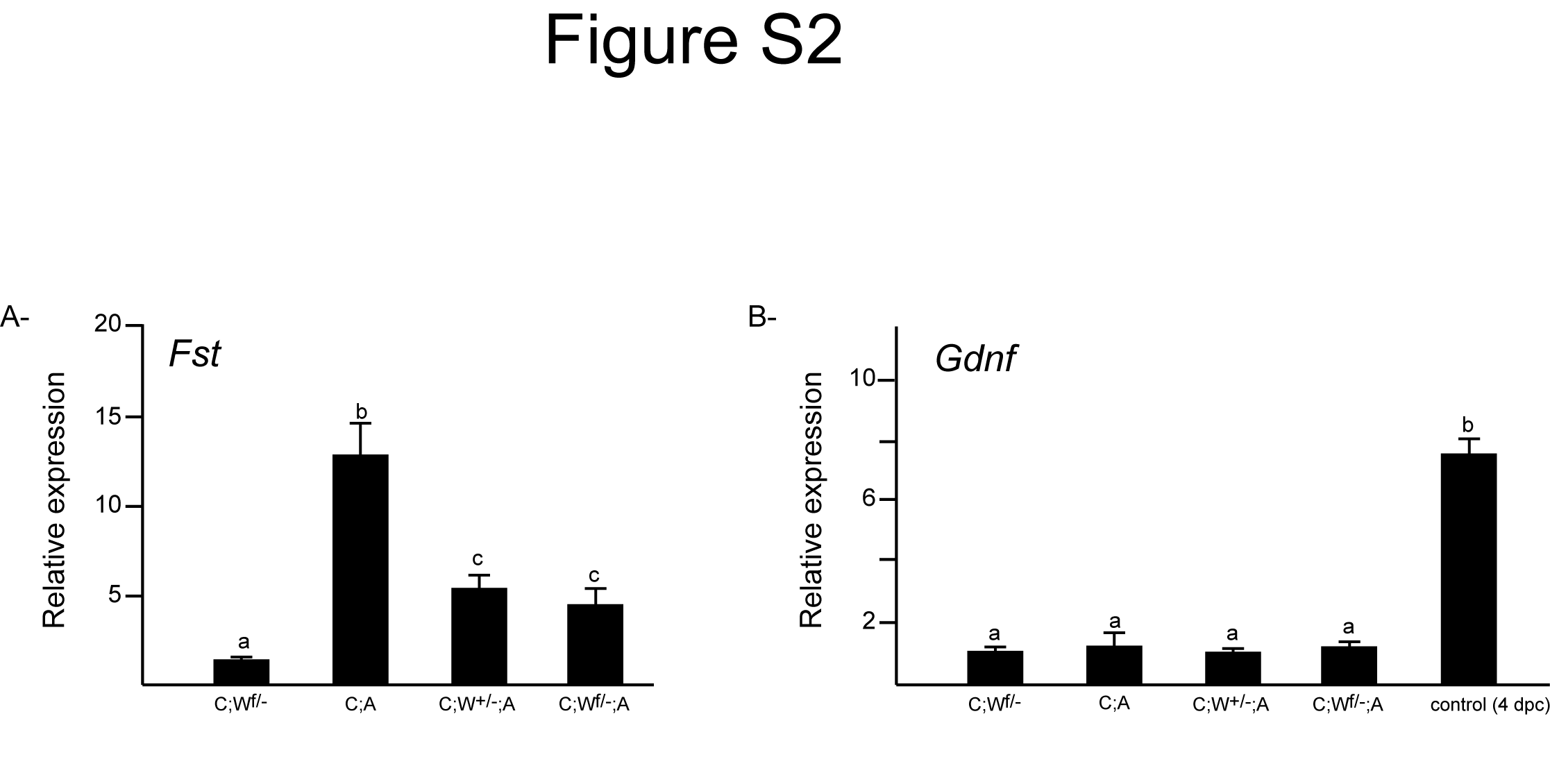

Supplement: Figure S2 — Fst and Gdnf expression in transgenic mouse testes. (A) Fst mRNA levels from 8 week-old animals of the indicated genotypes, n = 4 animals/genotype. C;Wf/−: Ctnnb1 tm1Mmt/+;Wnt4 flox/− (control), C;A: Ctnnb1 tm1Mmt/+;Amhr2 tm3(cre)Bhr/+, C;W+/−;A: Ctnnb1 tm1Mmt/+;Wnt4 +/−;Amhr2 tm3(cre)Bhr/+, C;Wf/−;A: Ctnnb1 tm1Mmt/+;Wnt4 flox/−;Amhr2 tm3(cre)Bhr/+. (B) Gdnf mRNA levels in the mice described in A. Gdnf expression was also evaluated in testes from 4 day-old wild-type mice, so as so confirm its physiological decrease during postnatal development. Data are expressed as mean (columns) ± SEM (error bars). Groups labeled with different letters (a, b, c) were significantly different (P<0.05). (TIF) [file pone.0029764.s002.tif]

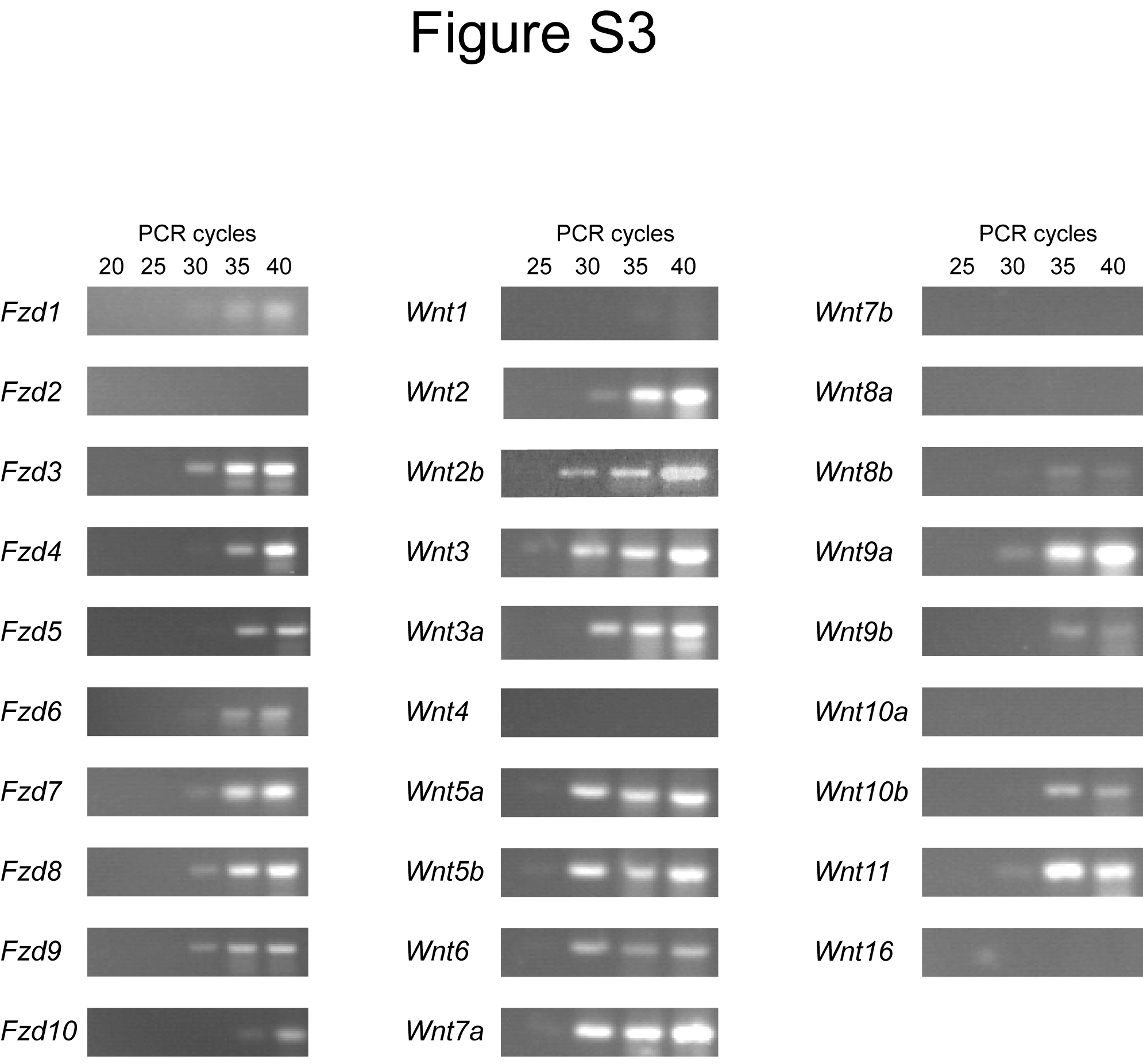

Supplement: Figure S3 — Analysis of the expression of Wnt and Fzd family members in the adult mouse testis. The expression of each gene was assessed by semi-quantitative RT-PCR analysis following the indicated numbers of PCR cycles. PCR products were separated by agarose gel electrophoresis, stained with ethidium bromide, and photographed under UV light. As shown, some degree of expression of all Wnt and Fzd genes was detected, with the exceptions of Fzd2, Wnt4, Wnt7b, Wnt8a, Wnt10a and Wnt16. (TIF) [file pone.0029764.s003.tif]
