# Supplementary material for: CTNNB1 Signaling in Sertoli Cells Downregulates Spermatogonial Stem Cell Activity via WNT4
Source: PLoS One. 2012 Jan 12;7(1):e29764. doi: 10.1371/journal.pone.0029764 (PMC3257228; doi:10.1371/journal.pone.0029764)
Supplement: Table S1 — Gene regulation in Sertoli cells in response to dominant-stable CTNNB1. Microarray analyses of cultured Sertoli cells from 3 week-old Ctnnb1 tm1Mmt/tm1Mmt mice infected for 24 h with adenoviruses to induce the expression of either Cre recombinase (Ad-Cre, to express dominant-stable CTNNB1) or eGFP (Ad-eGFP, control). (DOC) [file pone.0029764.s004.doc]

Table S1. Gene regulation in Sertoli cells in response to dominant-stable CTNNB1

| a) Genes upregulated by CTNNB1 | | |  | b) Genes downregulated by CTNNB1 | | |
| --- | --- | --- | --- | --- | --- | --- |
|  |  |  |  |  |  |  |
| Gene symbol | Fold upregulated | SD |  | Gene symbol | Fold downregulated | SD |
|  |  |  |  |  |  |  |
| TNFRSF22 | 4,71 | 0,13 |  | EGFP | 4,17 | 0,31 |
| ARRDC3 | 3,1 | 0,15 |  | BRP17 | 2,09 | 0,09 |
| GADD45B | 2,79 | 0,16 |  | ^b^ CBLN4 | 2,07 | 0,13 |
| GDF15 | 2,78 | 0,12 |  | HSPA1A | 2,05 | 0,5 |
| USP18 | 2,75 | 0,11 |  | GABABRBP | 1,97 | 0,05 |
| G1P2 | 2,51 | 0,1 |  | GAMT | 1,96 | 0,09 |
| RASSF1 | 2,44 | 0,05 |  | ^b^ RHOX8 | 1,96 | 0,1 |
| SGK | 2,21 | 0,15 |  | KCTD14 | 1,96 | 0,24 |
| SAA3 | 2,16 | 0,25 |  | GPRASP1 | 1,9 | 0,06 |
| ATP1B1 | 2,14 | 0,08 |  | ^b^ GSTM6 | 1,9 | 0,16 |
| MDM2 | 2,14 | 0,08 |  | ^b^ CST9 | 1,86 | 0,17 |
| GBP4 | 2,13 | 0,07 |  | RFWD3 | 1,86 | 0,09 |
| IFITM3 | 2,08 | 0,13 |  | ^b^ MMD2 | 1,85 | 0,09 |
| CXCL1 | 2,04 | 0,37 |  | DEFB19 | 1,84 | 0,2 |
| ANKRD1 | 1,99 | 0,15 |  | ^b^ AARD | 1,84 | 0,12 |
| PHLDA1 | 1,99 | 0,08 |  | HTATSF1 | 1,81 | 0,1 |
| PLK2 | 1,97 | 0,24 |  | SCARA3 | 1,79 | 0,07 |
| CDKN1A | 1,96 | 0,07 |  | DBP | 1,75 | 0,12 |
| PTX3 | 1,95 | 0,23 |  | FXYD6 | 1,73 | 0,07 |
| LCN2 | 1,94 | 0,15 |  | DIP3B | 1,71 | 0,07 |
| ODC1 | 1,94 | 0,07 |  | FOXQ1 | 1,69 | 0,09 |
| IGTP | 1,89 | 0,07 |  | RASL12 | 1,69 | 0,1 |
| PRKR | 1,88 | 0,1 |  | TNFSF13 | 1,69 | 0,09 |
| CCND1 | 1,83 | 0,05 |  | SH3GL2 | 1,69 | 0,02 |
| CYP1B1 | 1,8 | 0,25 |  | GPRASP2 | 1,69 | 0,07 |
| CEBPB | 1,78 | 0,09 |  | DDIT4L | 1,68 | 0,1 |
| OASL2 | 1,74 | 0,06 |  | CITED1 | 1,67 | 0,1 |
| ^c^ LMNA | 1,73 | 0,16 |  | BIRC2 | 1,67 | 0,1 |
| CXCL10 | 1,72 | 0,05 |  | SYAP1 | 1,67 | 0,11 |
| GADD45G | 1,68 | 0,09 |  | MMP11 | 1,66 | 0,11 |
| PHLDA3 | 1,68 | 0,11 |  | FBXO27 | 1,65 | 0,09 |
| TAGLN2 | 1,66 | 0,08 |  | KCNK13 | 1,64 | 0,12 |
| SPP1 | 1,66 | 0,18 |  | HIST1H2AO | 1,63 | 0,16 |
| ^b^ CAV1 | 1,66 | 0,27 |  | ZFP261 | 1,63 | 0,12 |
| ^c^ SLC19A2 | 1,66 | 0,22 |  | KCNA6 | 1,63 | 0,07 |
| TIMP1 | 1,65 | 0,07 |  | SNCB | 1,63 | 0,06 |
| EFS | 1,65 | 0,07 |  | PLEKHB1 | 1,62 | 0,13 |
| HOXB7 | 1,65 | 0,06 |  | TSPAN17 | 1,6 | 0,1 |
| FSCN1 | 1,64 | 0,1 |  | SYTL4 | 1,6 | 0,06 |
| GCH1 | 1,64 | 0,06 |  | PAK3 | 1,59 | 0,14 |
| PIP5K1B | 1,64 | 0,1 |  | ^a,b^ WT1 | 1,59 | 0,1 |
| LGALS3BP | 1,63 | 0,13 |  | BEX2 | 1,58 | 0,077 |
| RASL11B | 1,61 | 0,03 |  | QPCT | 1,58 | 0,05 |
| BICD2 | 1,6 | 0,05 |  | ZFP275 | 1,58 | 0,13 |
| CCL4 | 1,6 | 0,09 |  | ^b^ PPT1 | 1,57 | 0,14 |
| FKBP11 | 1,6 | 0,09 |  | PPP1R9A | 1,57 | 0,13 |
| NUPR1 | 1,6 | 0,09 |  | HIST1H2AK | 1,57 | 0,1 |
| ^a,b^ KLF4 | 1,59 | 0,03 |  | HIST1H2AG | 1,57 | 0,09 |
| BHLHB2 | 1,59 | 0,16 |  | VPS18 | 1,57 | 0,03 |
| EPHA2 | 1,59 | 0,12 |  | BEX4 | 1,56 | 0,1 |
| MCAM | 1,59 | 0,08 |  | ATP1B2 | 1,55 | 0,178 |
| BMPER | 1,59 | 0,16 |  | ST5 | 1,55 | 0,06 |
| NME7 | 1,59 | 0,06 |  | RHOBTB3 | 1,55 | 0,08 |
| TNFRSF12A | 1,58 | 0,14 |  | SLC30A2 | 1,55 | 0,12 |
| KLF5 | 1,58 | 0,08 |  | HIST1H2AD | 1,53 | 0,17 |
| IRGM | 1,58 | 0,27 |  | MYO7A | 1,53 | 0,22 |
| AXIN2 | 1,58 | 0,19 |  | DMRTC1A | 1,53 | 0,03 |
| CCNG1 | 1,58 | 0,1 |  | ^b^ SMARCA2 | 1,52 | 0,12 |
| PLAC8 | 1,57 | 0,09 |  | ^a,b^ SOX9 | 1,52 | 0,09 |
| HRAS1 | 1,57 | 0,1 |  | RNF144 | 1,51 | 0,06 |
| WFDC1 | 1,57 | 0,09 |  | ABHD4 | 1,5 | 0,21 |
| HERPUD1 | 1,56 | 0,04 |  |  |  |  |
| NPTX2 | 1,56 | 0,33 |  |  | | |
| ISGF3G | 1,56 | 0,09 |  |  | | |
| ^b^ HMGA1 | 1,56 | 0,08 |  |  | | |
| SFRS2 | 1,56 | 0,11 |  |  | | |
| IFIT3 | 1,54 | 0,08 |  |  | | |
| FEZ2 | 1,53 | 0,06 |  |  | | |
| SNRPF | 1,53 | 0,06 |  |  | | |
| ^c^ LGALS3 | 1,52 | 0,11 |  |  | | |
| TINAGL | 1,52 | 0,14 |  |  | | |
| TPBG | 1,52 | 0,07 |  |  | | |
| DDIT4 | 1,51 | 0,05 |  |  | | |
| HR | 1,51 | 0,06 |  |  | | |
| JUNB | 1,51 | 0,17 |  |  | | |
| SLCO2A1 | 1,51 | 0,07 |  |  | | |
| MYD116 | 1,5 | 0,03 |  |  | | |
| PDK4 | 1,5 | 0,16 |  |  | | |
| CFL1 | 1,5 | 0,05 |  |  | | |
| ^a,b^ FST | 1,5 | 0,06 |  |  | | |
| CRIP2 | 1,5 | 0,11 |  |  | | |
|  |  |  |  |  | | |

^a^ Gene associated with sex differentiation

^b^ Gene previously identified in differential expression screens between male and female gonads

^c^ Gene with known function in spermatogenesis
