# Supplementary material for: CTNNB1 Signaling in Sertoli Cells Downregulates Spermatogonial Stem Cell Activity via WNT4
Source: PLoS One. 2012 Jan 12;7(1):e29764. doi: 10.1371/journal.pone.0029764 (PMC3257228; doi:10.1371/journal.pone.0029764)
Supplement: Table S2 — Oligonucleotide primer sequences. List of primer sequences used for gene expression analyses by real-time RT-PCR. (DOC) [file pone.0029764.s005.doc]

Table S2. Oligonucleotide primer sequences.

Primer name Sequence (5’ to 3’)

Axin2-S CCACTTCAAGGAGCAGCTCAGCA

Axin2-AS TACCCAGGCTCCTGGAGACTGA

Cav1-S ACACCAAGGAGATTGCACCTGGT

Cav1-AS CTGATGCGGATGTTGCTGAATATC

Cbln4-S CACGGAACCGATCGTTCTGGAG

Cbln4-AS GGAGAAGTCGGGTTCTATAGAGGA

Fst-S CAAAGTCCTGTGAAGATATCCAGT

Fst-AS TAGGAAAGTTGTAGTCCTGGTCT

Gdnf-S GGTAAGAGGCTTCTCGAAGCGC

Gdnf-AS CCGATTCACAGGAACCGCTGCAA

Gstm6-S AGGTCATGGACACTCGAATTCAGA

Gstm6-AS GCATCCATGCATGGTCTTACTCA

Klf4-S CCTGCCAGACCAGATGCAGTCA

Klf4-AS GGTCACATCCACTACGTGGGAT

Rpl19-S CTGAAGGTCAAAGGGAATGTG

Rpl19-AS GGACACAGTCTTGATGATCTC

Sox9-S GACGTGCAAGCTGGCAAAGTTGA

Sox9-AS TGCTGCTTCGACATCCACACGTG

Wt1-S AGCTGTCCCACTTACAGATGCAT

Wt1-AS GGATGCTGGACTGTCTCCGTGT

Wnt4-S AGCTGTCATCGGTGGGCAGCAT

Wnt4-AS ACTGTCCGGTCACAGCCACACT
